# Serum apolipoprotein E may be a novel biomarker of migraine

**DOI:** 10.1371/journal.pone.0190620

**Published:** 2018-01-22

**Authors:** Naoki Yuasa, Eiichiro Nagata, Natsuko Fujii, Masatoshi Ito, Hideo Tsukamoto, Shunya Takizawa

**Affiliations:** 1 Division of Neurology, Department of Internal Medicine, Isehara Kyodo Hospital, Isehara, Japan; 2 Department of Neurology, Tokai University School of Medicine, Isehara, Japan; 3 Support Center for Medical Research and Education, Tokai University, Isehara, Japan; Università degli Studi di Milano, ITALY

## Abstract

Migraine attacks alter various molecules that might be related to the pathophysiology of migraine, such as serotonin, calcitonin gene-related peptide, and nitric oxide. The underlying pathophysiology of migraine is as yet unclear. We explored key proteins related to the pathogenesis of migraine here. Serum was collected from two patients with migraine with aura (MA) and seven patients with migraine without aura (MO) during attack-free periods and migraine attacks. Samples were analyzed using 2-dimensional gel electrophoresis. Nineteen protein spots were altered between the attack-free versus migraine attack periods. Mass spectrometric analysis was performed to identify the proteins within each of the 19 altered spots. Thirty-six proteins were significantly altered in samples collected during attack-free periods versus migraine attacks. The protein with the statistically most significant MASCOT/Mowse score (268±112) among lipoproteins was apolipoprotein (ApoE). In the MA and MO groups, ApoE protein levels were significantly higher during migraine attack than during the attack-free period (p<0.05). ApoE protein levels were also significantly increased in the MA group during the attack-free period compared to healthy controls and patients with tension type headaches (p<0.01). Migraine alters ApoE levels, especially in MA. ApoE might play an important role in the pathophysiology of migraine, and may act as a diagnostic biomarker of migraine.

## Introduction

Currently, the diagnosis of migraine is made in accordance with the criteria described in The International Classification of Headache Disorders 3 Beta (ICHD-3β)[1]. This diagnostic process is entirely symptom-based and therefore sometime poses a challenge to primary care physicians, especially for diagnosing chronic migraine [2–4]. The availability of reliable biomarkers for the diagnosis of migraine would provide a solution to such diagnostic ambiguity. Some previous reports have described biomarkers for migraine in peripheral blood and cerebrospinal fluid [5–7]. Polymorphisms in the serotonin transporter [8] and serotonin receptor type 2C genes differ between migraineurs and controls [9]. Moreover, Japanese patients with migraine with aura (MA) are more likely to carry point mutations within the methylenetetrahydrofolate reductase gene, which causes hyper-homocysteinemia [10]. In terms of vascular abnormalities, angiotensin-converting enzyme (ACE) gene deletion polymorphisms might lead to endothelial dysfunction and an increased susceptibility to migraine attacks. ACE deletion polymorphisms are more common in patients with migraine [11]. Moreover, the levels of α-fodrin mRNA, which encodes a cytoskeletal protein, are increased in MA [12]. However, the above-mentioned results have not been fully validated. With regard to biochemical markers of migraine, some papers have reported that the serum levels of calcitonin gene-related peptide (CGRP), a vasodilatation factor, are elevated in patients during migraine attacks compared with healthy controls [7,13]. The levels of ACE [14] and matrix metalloproteinase-9 [15] are also elevated in serum from migraineurs. Recently, reduced number and functioning of endothelial progenitor cells have been reported in migraineurs [16]. However, these biochemical markers are not specific to migraine. As genetic analysis entails sensitive ethical considerations, serum biomarkers seem to be more practical for diagnostic purposes. Hence, serum-derived biomarkers that are specific and relevant to migraine need to be identified. Ideally such biomarkers should confer important therapeutic clues as well.

Therefore, in the present study, we investigated protein alterations between attack and attack-free periods in migraineurs. Moreover, the proteins identified were evaluated for their potential to act as biomarkers for migraine.

## Materials and methods

### Participants

Seventy-four patients were carefully interviewed and examined, and diagnosis was made using the ICHD-3β [1].

The patient sera were prepared from peripheral blood samples obtained from the two patients with migraine with aura (MA) and seven patients with migraine without aura (MO) during an attack-free period in which the patients had had no attack for more than one week, and during migraine attacks. The demographics for these patients are shown in Table 1. Peripheral blood samples were also collected from a second set of patients with migraine (13 MA (average age: 34.7±10.3 years), 32 MO (35.3±10.1 years), seven with tension type headaches (TTH; 42.4±9.7 years), and thirteen age-matched healthy controls (40.5±11.0 years) during a pain-free period in which the patients had had no pain for more than one week. The ethical committee of the Tokai University School of Medicine, Japan approved the study, and all participants provided written informed consent (approval number: 14I-46).

**Table 1:** The two patients with migraine with aura (MA) (mean age 35.5 years) and seven patients with migraine without aura (MO) (32.3 years) were all women. All migraineurs had taken triptans during attacks, and three of the patients with MO were taking prophylactic medicines, such as anti-depressive drugs.

				Drugs			
Patient	Age	Gender	Headache type	Prophylaxis	Symptomatic	Mean of attack duration time (h)	Monthly frequency of attacks
1	42	F	MA	No	Triptan (Zolmitriptan)	24	2–3
2	29	F	MA	No	Triptan (Rizatriptan)	16	2
3	29	F	MO	No	Triptan (Sumatriptan)	24	2
4	49	F	MO	No	Triptan (Sumatriptan)	10	1
5	27	F	MO	No	Triptan (Rizatriptan)	15	2–3
6	29	F	MO	No	Triptan (Sumatriptan)	8	2–3
7	28	F	MO	Amitoriptyrine, Valproic acid, Topiramate	Triptan (Rizatriptan)	24	5–6
8	36	F	MO	Topiramate, Olanzapine, Propranolol	Triptan (Rizatriptan)	20	5–6
9	28	F	MO	Amitoriptyrine, Toparamate	Triptan (Rizatriptan)	15	1–2

### 2-dimensional gel electrophoresis (2-DE)

The serum samples were centrifuged at 3,000 r.p.m. for 15 min at room temperature. Afterward, albumin was removed from the serum samples using Protein A+G columns and Albumin and IgG removal kits (GE Healthcare, Germany). The eluted solution was precipitated with three volumes of acetone to one volume of sample at -20°C overnight. The samples were centrifuged at 18,000 r.p.m. for 10 min at room temperature. The supernatants were removed from the samples, and the samples were then dried out. The precipitates were diluted with rehydration buffer containing 30 mM Tris-HCl (pH 8.5), 7 M urea, 4% CHAPS, 2 M thiourea, 5 mM Mg (CH_3_COO)_2_, 2% ampholyte, 50 mM dithiothreitol (DTT). Isoelectric focusing (IEF) was carried out on 18 cm immobilized pH gradient (IPG) strips (pH 3–11) (Bio-Rad, USA). IPG strips were rehydrated overnight by loading the samples diluted with rehydration buffer containing 8 M urea, 2% CHAPS, 2% ampholyte, 50 mM DTT, and bromophenol blue (Merck, Germany). IEF was conducted at room temperature with a Mutiphor II system and a DryStrip kit (GE Healthcare, Germany). The running conditions were as follows: 500 V constant for 1h, 500–1000 V gradient for 2h, 1000–8000 V gradient for 3h, 8000 V constant for 3:45. The focused strips were equilibrated twice for 15 min in 10 ml equilibration solution at room temperature. The first equilibration was performed in a solution containing 6 M urea, 30% (w/v) glycerol, 2% (w/v) sodium dodecyl sulfate (SDS), 1% (w/v) DTT, and 50 mM Tris-HCl (Merck, Germany) buffer, pH 8.8. The second equilibration was performed in a solution with 2.5% (w/v) iodoacetamide (Merck, Germany). Separation in the second dimension was performed by sodium dodecyl sulfate polyacrylamide gel electrophoresis (SDS-PAGE) in a vertical slab of acrylamide (12% total monomer, with 2.6% cross-linker) using an Ettan DALTsix electrophoresis unit (GE healthcare, Germany). The proteins in the gels were visualized by staining with SYPRO Ruby (Molecular Probes, Eugene, OR, USA).

Gels were run in triplicate for each sample to obtain the best spot resolution. Gel images were acquired using a FluoroPhoreStar 3000 image capture system (Anatech, Tokyo, Japan) and analyzed using the PDQuest 2-D analysis software program (version 8.0, Bio-Rad, CA, USA). This software compares bi-dimensional gel images to reveal increased or decreased protein bands. After matching, all gel spots were normalized by local regression model (LOESS) method using PDQuest 2-D analysis software (Bio-Rad, Hercules CA).The results for the spots are shown in Table 2 calculated as the ratio between the spot intensity values in the attack-free period or the attack period in samples from migraineurs.

**Table 2:** We identified 19 spots that were significantly differentially expressed between attack and attack-free periods in migraineurs. ApoE was detected in four of the spots.

Spot No.							
**2**	APO C-III	Vitronectin					
**15**	APO C-III	Vitronectin					
**18**	APO C-II	APO C-III					
**1007**	Haptoglobin						
**1802**	SERPINA1	IGHA1	SERPINC1	SERPINA7	IDH3A	AGT	Ig heavy chain V-III region
**3120**	Haptoglobin	Haptoglobin-ralated protein					
**3507**	**APOE**	Transthyretin	Complement factor B				
**3510**	**APOE**	CFB	ALB				
**4205**	APOA1						
**4402**	Haptoglobin	Complement C3	Serum amyloid P-component	Haptoglobin-related protein	Ig mu chain C region	Serum amyloid P-component	
**4506**	**APOE**	APOA1	Serum amyloid P-component	ALB	Ig lambda-2 chain C regions	Haptoglobin	Sex hormone-binding globulin
**5004**	Transthyretin	Calmodulin-like protein 5	Suprabasm	Prolin-rich protein 22			
**5111**	Mannan-binding lectin serine protease 2	ALB	APOA1				
**5202**	GPX3	APOA1					
**5304**	Ig lambda-2 chain C regions	Ig kappa chain C region	Alpha-1-antitrypsin	Ig lambda chain V-III region			
**5503**	TTR	**APOE**	ALB	Serum amyloid P-component			
**6007**	Serum amyloid A-1 region						
**7201**	Ig kappa chain V-III region	Ig kappa chain C region	Ig kappa chain V-I region	Ig kappa chain V-III region (fragment)	Ig lambda-2 chain C regions		
**8108**	Serum amyloid A-1 region						

APO C-II: apolipoprotein C-II; APO C-III: apolipoprotein C-III; SERPINA1: α-1-antitrypsin; IGHA1: Ig α-1 chain C region; SERPINC1: antithrombin-III; SERPINA7: thyroxine-binding globulin; IDH3A: isocitrate dehydrogenase (NAD) subunit αmitochondria; AGT: angiotensinogen; APOA1: apolipoprotein A-1; CFB: complement factor B; ALB: serum albumin; GPX3: glutathione peroxidase 3; TTR: transthyretin.

### Tandem mass spectrometry (MS/MS) and protein identification

Protein identification in gel spots was performed using an LCMS-IT-TOF instrument (Shimadzu, Kyoto, Japan). The gel pieces of interest were excised from the gel, and in-gel digestion of proteins was performed. The gel pieces were washed with 30% acetonitrile in 50 mM NH_4_HCO_3_ and then with 60% acetonitrile in 50 mM NH_4_HCO_3_. Then, the samples were dried using a SpeedVac concentrator (Thermo Fisher Scientific, MA, USA). For in-gel digestion of proteins, the samples were incubated with a trypsin solution at 37°C overnight. Extracted trypsinized peptides were eluted in a stepwise manner with 30%, 100%, and 0% of acetonitrile in 50 mM NH_4_HCO_3_. The samples were dried and then dissolved in 50 mM NH_4_HCO_3_.

Tryptic peptides were separated via reversed-phase liquid chromatography/mass spectrometry using Prominence nanoLC (Shimadzu, Kyoto, Japan) for analytical separation on a PicoFrit^TM^ BetaBasic C18 column (100 mm × 75 μm; New Objective, Wohsburn, MA, USA). Mass spectrometric analysis ([+] electrospray ionization) was carried out on an LCMS-IT-TOF instrument with argon gas for ion cooling and collision induced dissociation experiments. Tandem mass spectrometry data were obtained in a data-dependent manner. A Mascot search engine (Matrix Science, Boston, MA, USA) was used for protein database searching. Proteins with a statistically significant MASCOT/Mowse score (> 28), indicating identity or extensive homology (p < 0.05), were considered for identification.

### ELISA assay for ApoE

Based on the results from the experiments described above, we focused on ApoE for ELISA assay. The sera were centrifuged at 3,000 r.p.m. for 15 min at room temperature. The supernatants were collected, and the samples were quickly frozen for -80°C storage. Those samples were measured with an enzyme-linked immunosorbent assay (ELISA) for ApoE (abcam, Cambridge, UK).

### Statistics

To determine the statistical differences between groups, one-way ANOVA analysis was applied. Values are given as mean±standard deviation of the mean (SD). Statistical analysis was done with SPSS. Statistical significance was accepted at p<0.05.

## Results

### Protein identification

**Fig 1:**
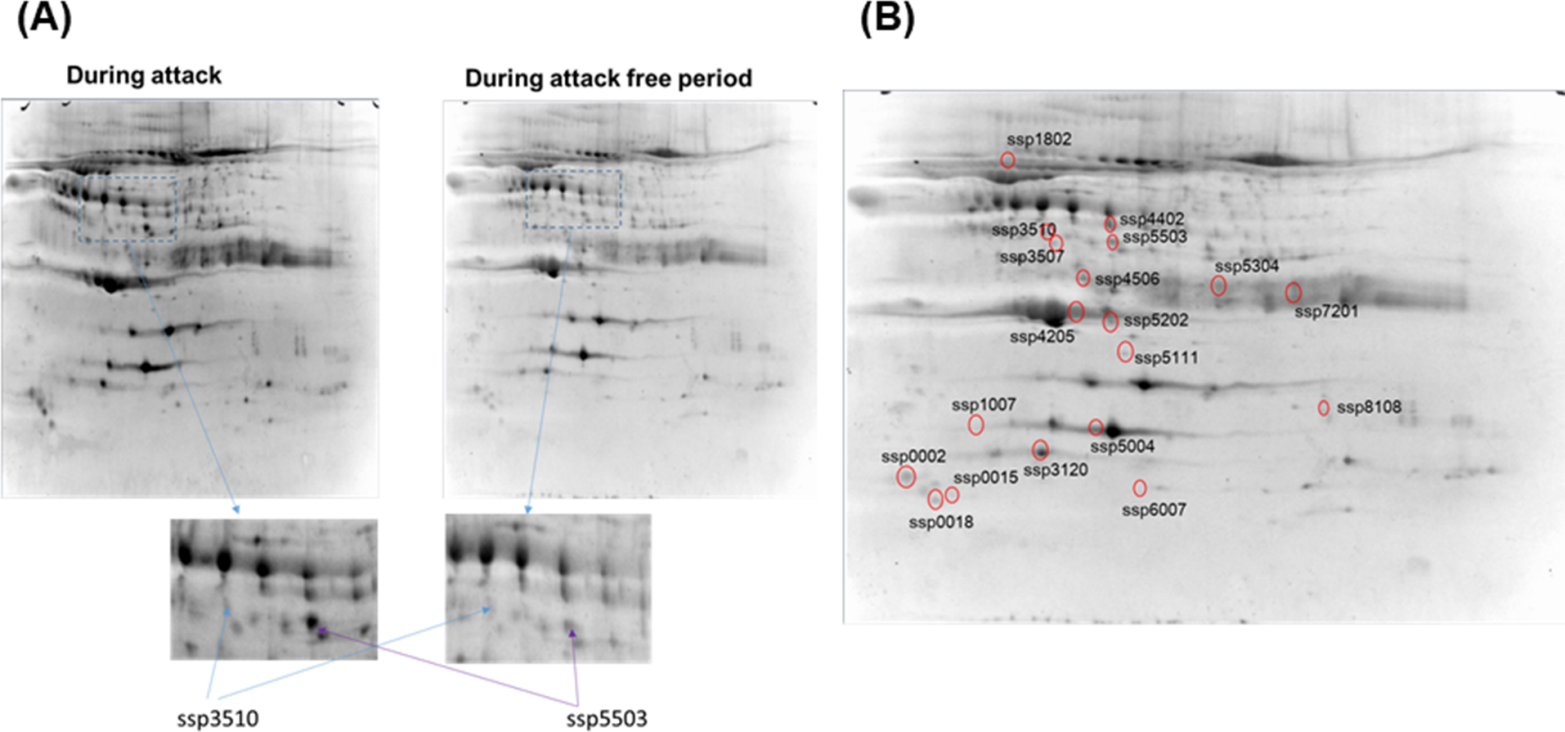
Representative two-dimensional electrophoresis analysis of samples collected during migraine attacks. Gel images are shown for samples from migraine attack and attack-free periods after analysis and normalization using PDQuest software. The arrows on the spots with identical spot numbers across gels indicate proteins at the same gel positions. (A) Representative spot ssp3510 contained APOE, complement factor B, and albumin, ssp5503 contained transthyretin, APOE, albumin, and serum amyloid P-component. (B) 19 spots obtained from the attack period gel showed over 3-fold differences in densitometric volumes in the attack period gel compared with those in the attack free period gel (Table 2).

**Fig 2:**
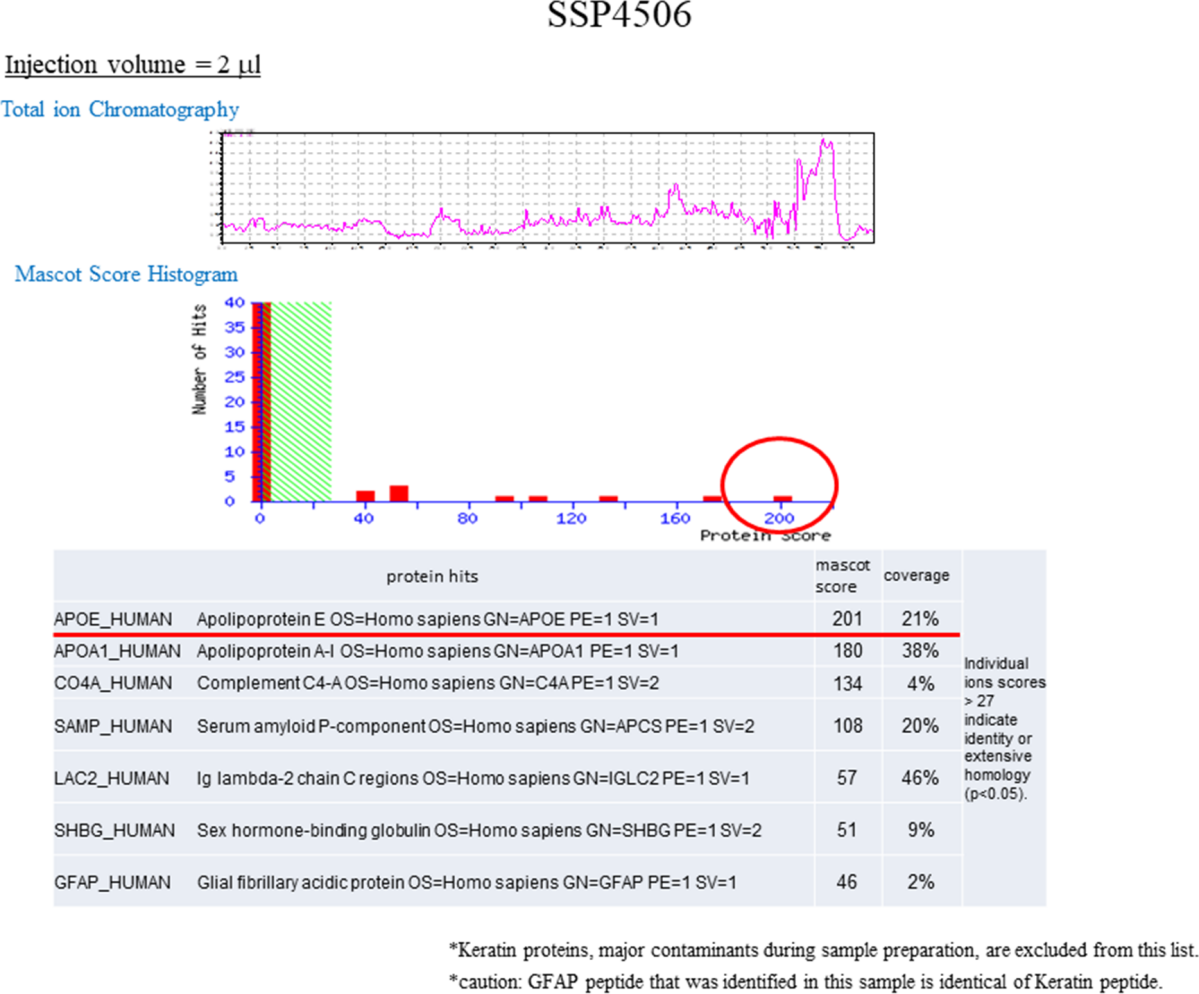
Representative Tandem mass spectrometric analysis of a 2-DE gel spot (SSP4506) showing statistically significant differences in spot intensity between the attack-free period and the attack period of migraineurs. (Upper panel) Total Ion Chromatogram of tryptic digest from SSP4506. (Middle panel) Mascot histogram of the score distribution for the proteins identified in SSP4506. (Lower Table) ApoE and another six proteins were identified as having significant Mascot scores (>28) in SSP4506.

Fig 1 shows the SYPRO Ruby-stained 2D gels of cell lysates collected from patients in the MA and MO groups during attack-free periods and migraine attacks. Based on the experimental pI and mass determined, we obtained 19 spots from the attack period gel that showed over 3-fold differences in densitometric volumes in the attack period gel compared with those in the attack free period gel. The proteins obtained by in-gel trypsin digestion of the spots were identified (Fig 2 and Table 2). The protein with the statistically most significant MASCOT/Mowse score among lipoproteins was ApoE (Table 3). Therefore, we investigated ApoE in further experiments described below.

**Table 3:** Note that ApoE had the highest score of all lipoproteins in Table 2.

APO E	268±112
APO C-II	158
APO C-III	73±17.0
APO AI	160±158

### Validation of ApoE by ELISA assay

**Fig 3:**
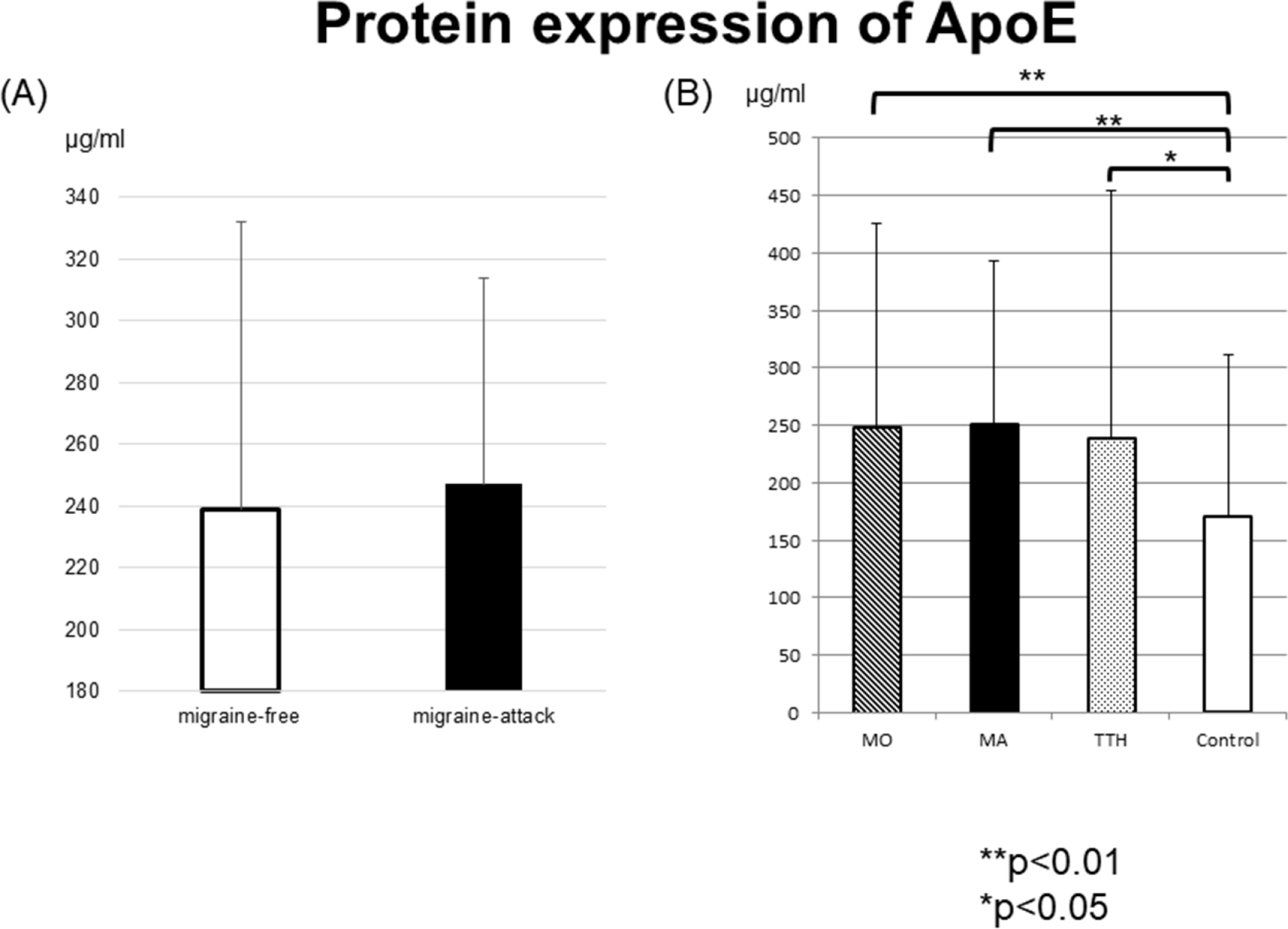
ApoE protein levels in sera of patients with migraine (MA and MO), TTH, and healthy controls. (A) The levels of ApoE protein in samples collected during migraine attacks tended to be higher than those in the samples collected during the attack-free periods. (B) ApoE protein levels in the MA, MO, and TTH groups were significantly higher compared to those in healthy controls (p<0.05). Notably, in the MA and MO groups during the attack-free period, ApoE protein levels were more significantly higher compared to those in healthy controls (p<0.01), while there was no significant difference in ApoE protein levels between the MA, MO, and TTH.

We identified ApoE as a candidate biomarker for migraine. The levels of ApoE protein in sera from the MA, MO, TTH, and healthy control groups were examined using ELISA assay. We first determined that the level of ApoE protein during the attack period in the migraineurs (MA and MO groups) tended to be higher than that during the attack-free period (Fig 3). Moreover, during the attack-free period, ApoE protein levels in the MA, MO, and TTH groups were significantly higher compared to those in healthy controls (p<0.05). Notably, in the MA and MO groups during the attack-free period, ApoE protein levels were more significantly higher compared to those in healthy controls (p<0.01), while there was no significant difference in ApoE protein levels between the MA, MO, and TTH (Fig 3(A) and 3(B)).

## Discussion

In the present study, we demonstrated that our proteomics-based approach is useful for screening serum biomarkers relevant to migraine attacks. Our analysis revealed that serum ApoE protein can serve as a novel biomarker that reflects the pathogenesis of migraine.

ApoE is well known as one of the key molecules involved in Alzheimer’s disease and other neurological disorders. ApoE is a 34 kDa glycoprotein and is a major determinant of lipid transport and metabolism [17,18]. Plasma levels of ApoE and other lipids and lipoproteins are under strong genetic influence of the ApoE polymorphism that defines the three ApoE isoforms [19]. Moreover, the ε4 allele is a strong genetic risk factor for Alzheimer’s disease [20]. However, many previous studies have reported that the ApoE polymorphism is not associated with migraine [21–23]. ApoE is profoundly up-regulated after injury, especially sciatic nerve crush injury where it has been found to increase several hundred-fold [24,25].

The pathophysiology of migraine is known to depend on the activation and sensitization of the trigeminovascular pain pathway, and cortical spreading depression (CSD) is the neurophysiological correlate of migraine aura [26]. CSD is a long-lasting suppression of neuronal activity preceded by a slowly propagating wave of neuronal and glial depolarization accompanied by massive ion fluxes in the cerebral cortex. It coincides with, and is thought to underlie, migraine aura. Several molecules, such as CGRP, serotonin, and glutamate fluctuate in CSD conditions. Moreover, oxidative stress-related genes, such as glutathione-S-transferase-5 and ApoE, are induced by CSD [27]. However, it is unclear what alterations occur during migraine attacks.

During CSD in the cerebral cortex, the inflammation resulting from the release of inflammatory mediators, such as cytokines derived mainly from microglia, may increase further [28,29]. On the other hand, in the dura mater mast cells release inflammatory mediators and sustain the activation and sensitization of meningeal nociceptors during migraine attacks [30]. Cells, such as endothelial cells, mast cells, microglia, and neurons, are also involved in the synthesis of nitric oxide (NO), which modulates neurotransmission in the central nervous system. NO is known to be associated with the pathophysiology of migraine. NO contributes to vasodilatation, increased local blood flow, and decreased vascular resistance in the cerebral circulation, which is important for pain perception to be transmitted through the trigeminal neurons. Since NO is released as part of the response to tissue inflammation and injury, ApoE-mediated regulation of NO may be an important link in the tissue inflammatory process [31]. An inflammatory response may promote and sustain the activation of meningeal nociceptors during a migraine attack.

**Fig 4:**
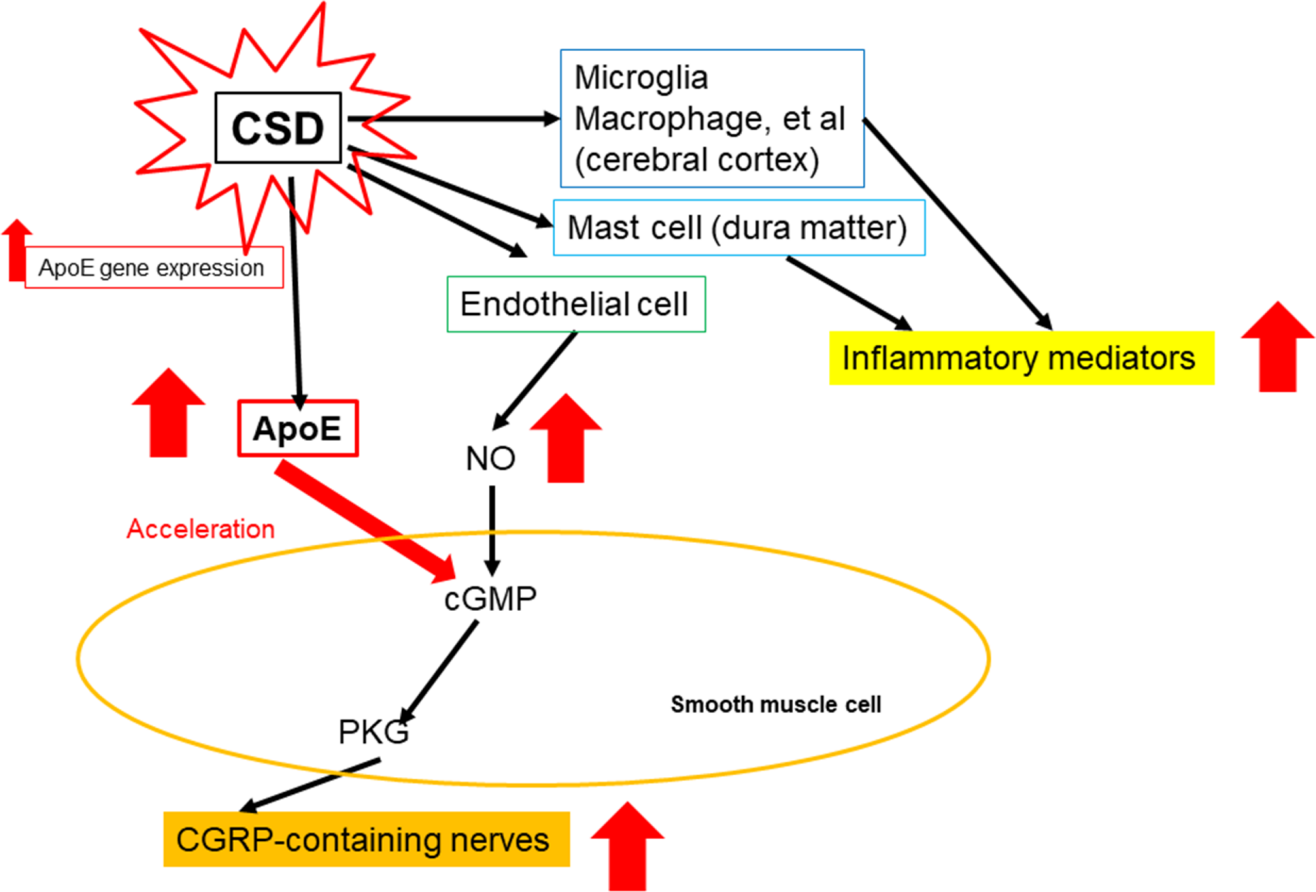
Schema of alterations in ApoE levels induced by CSD. During CSD, several inflammatory mediators are released from microglia, macrophages, and mast cells. Simultaneously, nitric oxide (NO) is released from endothelial cells. NO activates cyclic GMP (cGMP) in smooth muscle cells. Then, cGMP activates protein kinase G (PGK), and, finally, calcitonin gene-related peptide (CGRP)-containing nerves are activated. When NO is generated, ApoE accelerates the activity of cGMP. CSD also induces ApoE gene expression. This figure is modified from cited material (references([32], [33]).

ApoE-deficient mice have a smaller number of CGRP-containing nerves than wild-type mice [32,33]. This indicates that ApoE is associated with CGRP. It is also well known that CGRP plays an important role in the pathophysiology of migraine. These findings indicate that ApoE might be a key molecule in the pathophysiology of migraine (Fig 4).

In conclusion, the relationship between the pathophysiology of migraine and ApoE has been unclear to date. ApoE might indirectly play an important role in the pathophysiology of migraine. Moreover, ApoE might be a useful biomarker of migraine, especially in patients with MA based on our results.
